# Replication associated nuclear DNA mismatch repair across kingdoms

**DOI:** 10.1042/BST20260765

**Published:** 2026-06-25

**Authors:** Claudia P. Spampinato, Julieta Giri

**Affiliations:** Centro de Estudios Fotosintéticos y Bioquímicos (CEFOBI), Facultad de Ciencias Bioquímicas y Farmacéuticas, Universidad Nacional de Rosario, Suipacha 531, 2000 Rosario, Argentina

**Keywords:** DNA synthesis and repair, genome integrity, mutation

## Abstract

The mismatch repair (MMR) system is an essential DNA repair mechanism that recognizes and corrects single base–base mismatches and unpaired nucleotides that escaped the proofreading exonuclease activity of DNA polymerases or recombination intermediates. This pathway is highly conserved throughout evolution. However, the nature and number of MMR proteins differ between eukaryotes and prokaryotes. Even more, the plant MMR system contains an ancient duplicated MMR protein. In addition, developmental processes vary among eukaryotic organisms. One striking feature is plant genome stability maintenance over multiple generations because embryogenesis and seed development occur after many divisions during plant vegetative growth. Thus, it was of our interest to review the present state of knowledge with respect to the MMR mechanism from eukaryotic organisms, with special comparisons between human, yeast, and plant systems.

## Introduction

DNA is continuously vulnerable to nucleotide sequence alterations and damages from endogenous and environmental factors [1]. Nucleotide sequence alterations can arise during DNA synthesis and include the incorporation of a non-complementary Watson–Crick base or the misalignment of the two DNA strands at microsatellites (short repetitive sequences). Nucleotide sequence changes can also occur from spontaneous base deamination. DNA damages can be induced by reactive oxygen species, UV- and ionizing-radiation and alkylating agents. DNA damages comprise abasic sites, oxidized bases, pyrimidine dimers, DNA adducts, inter- and intra-strand crosslinks, and single- and double-strand breaks. These DNA errors and lesions are repaired by multiple and partially overlapping DNA repair mechanisms [2]. One of these is the DNA mismatch repair (MMR) system, an evolutionary conserved pathway throughout species. Provided here is a review of major answers to eight questions about the MMR system from eukaryotic organisms, with a special focus on human, yeast, and plant MMR initiation factors, mainly collected over the last years.

## What does the MMR system do?

The MMR system is best known for its role in the recognition and correction of non-Watson–Crick base pairs and unpaired nucleotides arising during DNA replication and DNA recombination between non-identical DNA sequences [2]. In addition to improving the fidelity of DNA replication and recombination, the MMR system is also implicated in the response to helix-distorting, oxidative, methylated, and interstrand crosslink DNA lesions [3,4].

## How does the MMR system function?

The MMR system functions in sequential steps. Genes IDs from *Saccharomyces cerevisiae, Homo sapiens*, and *Arabidopsis thaliana* that codify core eukaryotic MMR nuclear proteins are shown in Table 1.

**Table 1:** Identified genes involved in nuclear MMR

Gene	*S. cerevisiae*	*H. sapiens*	*A. thaliana*
*MSH2*	854063	4436	821383
*MSH3*	850454	4437	828659
*MSH6*	851671	2956	828147
*MSH7*	–	–	822040
*MLH1*	855203	4292	826493
*PMS1*	855642	5378 (*MLH2*)	827997
*PMS2*	–	5395	–

*S. cerevisiae, H. sapiens*, and *A. thaliana* gene IDs were retrieved from https://www.ncbi.nlm.nih.gov/. Human MLH2 is the homolog of yeast Pms1, which heterodimerizes with MLH1 to form MutLβ.

**Figure 1:**
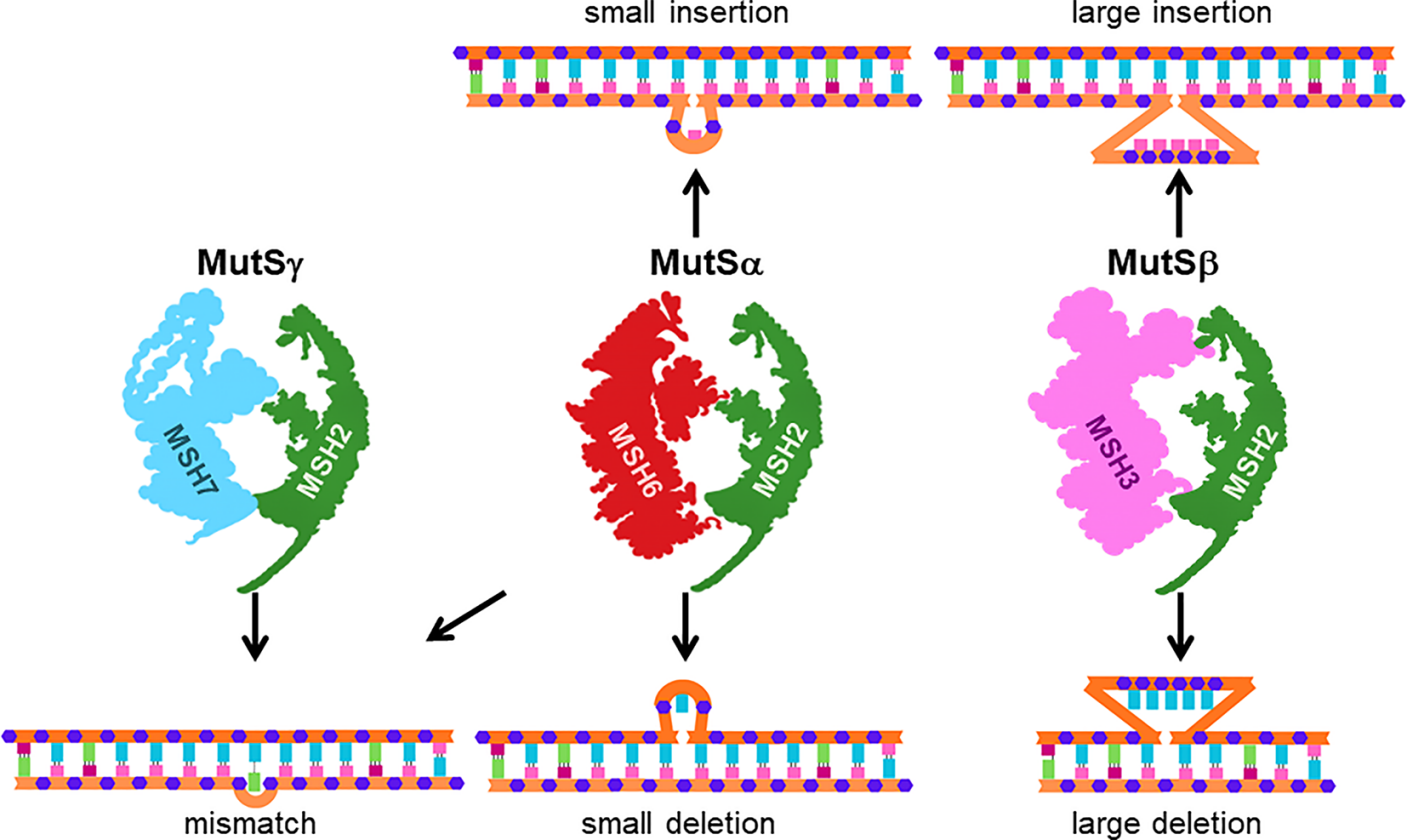
Schematic representation of DNA lesion recognition by MSH proteins MSH2 (green) interacts with MSH7 (light blue). MSH6 (red) and MSH3 (pink) to form MutSγ, MutSα, and MutSβ heterodimers, respectively. MutSγ, recognizes certain mismatches; MutSα, mismatches and small unpaired nucleotides; and MutSβ, large unpaired nucleotides. Parental and daughter strands of a DNA segment are shown in dark and light orange, respectively; adenine, thymine, cytosine, and guanine, in light blue, pink, burgundy, and green boxes, respectively; 5′ phosphate and phosphodiesters, in violet hexagons; hydrogen bonding of A-T and C-G base pairs, with two or three connecting lines, respectively.

Step 1: DNA lesion recognition by MutS homolog (MSH) proteins. Among these proteins, MSH2, MSH3, MSH6, and MSH7 are involved in nuclear MMR activity. MSH2 interacts with MSH6, MSH3, and MSH7 to form MutSα, MutSß, and MutSγ heterodimers, respectively (Figure 1). MutSα recognizes mismatches and unpaired nucleotides [insertion/deletion loops (IDLs) of 1–2 nucleotides]; MutSß, larger IDLs; and MutSγ, certain mismatches [2]. MutSα is present in many eukaryotes; MutSß, in some eukaryotes but missing from ecdysozoan and lophotrochozoan genomes; and MutSγ, only present in plants [5,6].

Step 2: DNA repair complex assembly. Mismatch-bound MutS proteins recruit MutL heterodimers to form an MMR initiation complex in the presence of ATP. MutL homolog 1 (MLH1) interacts with PMS1 (yeast and plant homolog) or PMS2 (human homolog) to form MutLα, the primary MutL complex in MMR [7]. MLH1 also interacts with MLH2 and MLH3 to form MutLß and MutLγ, respectively. These heterodimers play minor roles in human MMR system but are implicated in meiotic recombination [7]. In *S. cerevisiae*, MutLß acts as a non-essential protein that stimulates MMR activity while MutLγ resolves meiotic recombination intermediates [8,9]. Similarly, *A. thaliana* MutLγ is involved in crossover formation, but so far there are no reports of *A. thaliana* MutLß [10].

It should be noted that in human cells (i) the MMR initiation complex contains more than one MutLα molecule flanking MutSα bound to a mismatch, (ii) MutLα can be recruited to a strand break by interaction with proliferating cell nuclear antigen (PCNA), and (iii) MutLα (or MutLα/PCNA) at a mismatch and MutLα at a strand break interactions bring the mismatch into proximity with the strand break via a loop-mediated mechanism [11].

Step 3: Cleavage of the mismatch-containing daughter DNA strand. MutLα is an endonuclease that upon activation by PCNA introduces 5′ nicks into the discontinuous DNA strand near the mismatch [11–13].

Step 4: Removal of the mismatch-containing DNA section. There are two subpathways. One subpathway is dependent on the activity of exonuclease 1 (EXO1) that excises DNA in a 5′ to 3′ direction towards the mismatch [11,14]. Once the excision reaches the MutSα– or the MutSα–MutLα-mismatch complexes, MutSα or MutSα–MutLα proteins slide away from the mismatch allowing EXO1 to remove the error [11]. Nucleotide removal termination depends on MutLα and EXO1 physical interactions. The other excision subpathway is independent on EXO1, but dependent on other nucleases [Rad27 in *S. cerevisiae*; or flap endonuclease (FEN1), FANCD2-associated nuclease 1 (FAN1), or DNA2 in mammalian cells] [14–17].

Step 5: MMR termination. In the EXO1-dependent subpathway, a replicative DNA polymerase (DNA polymerase δ or ε) fills the single stranded DNA gap generated in the presence of PCNA and the single-stranded DNA binding protein replication protein A [11]. DNA ligase I completes the repair. In the EXO1-independent subpathway, the 5′-nick generated by MutLα marks an initiation point for DNA polymerase δ, which performs a strand displacement synthesis [11,15]. Single-stranded DNA tails are cleaved by Rad27 or DNA2. DNA ligase I ligates the nick.

**Figure 2:**
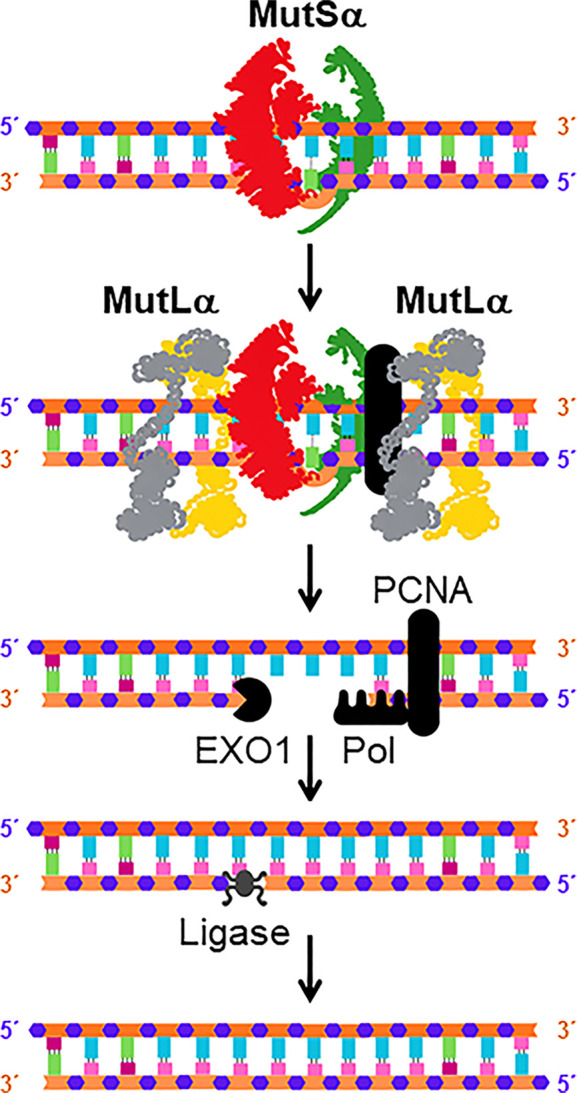
Schematic representation of the recognition and correction of DNA lesions by the MMR system Step 1: DNA lesion recognition by MutSα (or MutSβ or MutSγ). Step 2: Assembly of the DNA repair complex containing the MutS protein, MutLα, and PCNA. Step 3 (not shown): Activation of the MutLα endonuclease activity and cleavage of the mismatch-containing daughter DNA strand. Steps 4 and 5: Removal of the mismatch-containing DNA section by the EX01 subpathway; resynthesis of the DNA by a replicative DNA polymerase (Pol) and nick ligation by the DNA ligase I (Ligase).

A schematic representation of the major steps in the DNA repair process by the MMR system is shown in Figure 2.

## Which is the origin of the eukaryotic MMR system?

The origin of nuclear MSHs can be traced back to the Asgard archaea (greater than 2000 million years ago) [18]. This orthologous relationship between an archeal ancestor and nuclear MMR initiation factors was also observed as a monophyletic clade in the evolutionary tree [19]. Two things should be noted. First, MSH7 was assigned as another eukaryote subfamily [19]. Second, MSH3 is missing in several species [5,19].

Nuclear MLHs were also associated with Asgard archaea [18]. However, analysis of the MLH1 linker motif showed conserved sequence patterns in eukaryotes that were absent in Asgard archaea, among other prokaryotes [20]. The linker domain along with the N-terminal ATP binding domain and the C-terminal domain, which could also contain an endonuclease motif, are the three structurally conserved regions in MLH family proteins [21]. The pattern of branching of the phylogenetic tree of Mlh1 and Pms1/Pms2 homologs, among others, from the unicellular eukaryote *Dictyostelium discoideum*, the mushroom *Coprinopsis cinerea*, the budding yeast *S. cerevisiae, H. sapiens*, and *A. thaliana* and showed two main groups: the Mlh1 family and an ancestor that then divided into the Pms1/Pms2 family, among others [21]. Thus, MLH gene duplication and specialization occurred before the divergence of standard model organisms [20,21].

## How is the connection between the chromatin structure and the MMR system?

Chromatin is the nucleoprotein complex of DNA and histone proteins [22,23]. There are five types of histones (H1, H2A, H2B, H3, and H4). Two each of H2A, H2B, H3, and H4 histone proteins form the nucleosome core around which DNA is wrapped [24] while H1 histone protein interacts with the region of DNA between core particles [25].

Chromatin accessibility is regulated by remodeling proteins through two major mechanisms [26]. First, histones can undergo post-translational modifications such as methylation, acetylation, and phosphorylation, among others, of several amino acid residues including K, R, S, T, and Y [27]. These post-translational modifications are added, removed, or read by writers, erasers, or readers, respectively [28]. Histone reader domains recognize specific modifications and then mediate downstream responses. MSH6 contains a PWWP or a Tudor histone reader domain in metazoa or land plants, respectively, that recognizes epigenomic marks in coding regions of active genes (H3K36me3 or H3K4me1, respectively) and therefore contributes to a reduction in mutation rates in those regions of the genome (Table 2; [29,30]).

**Table 2:** Connection between the chromatin structure and the MMR system in model organisms

		MMR system						References
		*S. cerevisiae*		*H. sapiens*		*A. thaliana*		
**Chromatin structure**	**Histone modifications**		Reader domain absent in Msh6	**H3K36me3**	PWWP domain of MSH6	**H3K4me1**	Tudor domain of MSH6	[29,30]
	**Chaperones**	**CAF-1**	Mlh1 Msh2	**CAF-1**	MutSα	**CAF-1**	?	[31,32]
	**Remodelers**	**Fun30**	Msh2	**SMARCAD1**	MSH2 MLH1	**CHR19**	None	[33–35]
		**Rad5**	Msh2 Mlh1	**HLTF/SHPRH**	MSH2 (HLTF) MLH1 (SHPRH)	**RAD5A**	?	[36]
		**Swi1**	?	**ARID1A**	MSH2	**LFR**	?	[37]

The question mark (?) represents unknown interactions. Details are described in the text.

Second, chromatin accessibility and nucleosome positioning can be altered by chaperones and ATP-dependent chromatin remodelers. These multiprotein complexes have been shown to play an important role in MMR (Table 2). The chromatin assembly factor-1 (CAF-1) chaperone is a nuclear complex involved, with the assistance of ASF1A, in the deposition of histone (H3–H4)_2_ tetramer onto DNA during DNA replication and DNA repair [38]. Biochemical evidence suggests that human MutSα inhibits CAF-1-mediated chromatin assembly at and near a mismatch [31]. Once the mismatch is repaired, the DNA is packaged into nucleosomes. In addition, CAF-1-mediated chromatin assembly controls the degradation of the discontinuous daughter strand during MMR [31]. In *S. cerevisiae*, CAF-1 was shown to suppress the cytotoxic activity of the MMR system to a DNA methylating agent [32]. In *A. thaliana*, CAF-1 loss impacted on the expression of genes from some DNA repair pathways [39], although so far no reports exist on the interaction between CAF-1 and MMR proteins.

ATP-dependent chromatin remodelers belong to one of the four families [switch/sucrose-non-fermenting (SWI/SNF), imitation switch, chromo-domain helicase DNA-binding, and inositol requiring 80 (INO80) families] of the DNA helicase superfamily 2 [26]. SMARCAD1 (SNF2 related chromatin remodeling ATPase with DExD box 1) is an SNF2 enzyme that interacts with MSH2 under normal growth conditions and, provided that the ATPase activity of SMARCAD1 is not impaired, SMARCAD1 also assists in MLH1 recruitment on damaged chromatin in human cells [33]. Fun30, the *S. cerevisae* homolog of SMARCAD1, was also reported to cooperate with the Msh2-dependent MMR [34]. Similar results were obtained by the same authors when using nucleoplasmic extracts from *Xenopus laevis* eggs [34]. The *A. thaliana* homolog of SMARCAD1/Fun30 is the CHROMATIN REMODELING19 (CHR19) [35]. However, CHR19 appears to have lost the specific role in DNA repair [35].

The helicase-like transcription factor (HLTF) and SNF2 histone linker PHD RING helicase (SHPRH) are members of the SWI/SNF2 family of chromatin remodelers [40,41]. HLTF constitutively interacts with MSH2 under basal conditions, while SHPRH with MLH1 only during S-phase [42]. HLTF–MSH2 interaction is also involved in suppressing G-quadruplexes accumulation [43]. Rad5, the *S. cerevisae* homolog of HLTF and SHPRH, interacts with both Msh2 and Mlh1 [42]. RAD5A, the *A. thaliana* homolog of the three proteins HLTF, SHPRH, and Rad5, play a role in DNA damage response [44,45], but the involvement of RAD5A in plant MMR has not still been reported.

The ARID1A (AT-rich interaction domain 1A), also known as BAF250a, is a subunit protein of the SWI/SNF chromatin remodeling complex. ARID1A recruits MSH2 to chromatin during human MMR activity [37]. Consistently, ARID1A deficiency compromises MMR activity and enhances mutagenesis [37]. Yeast *SWI1* and leaf- and flower-related (*LFR*) genes are considered homologs of human ARID1A [46]. However, so far involvement of Swi1 and LFR in MMR has not yet been characterized.

All these observations indicate that chromatin accessibility play important roles in MMR activity.

## Where is the MMR system expressed?

The core proteins of the mammalian MMR system are thought to be operational in all cell types, including oocytes, sperm and embryos [47,48], but is preferentially expressed in proliferating cells [49–51]. Accordingly, the core factors of the plant MMR system are also preferentially expressed in proliferating tissues [2]. *MSH6* and *MSH7* expression was strong in *A. thaliana* shoot and root apical meristems and floral organs but undetectable in mature leaves and *MSH3* expression was clearly restricted to *A. thaliana* flowers [52–54]. These results confirm the essential contribution of the MMR system towards genome maintenance in rapidly dividing tissues.

## When is the MMR system expressed?

The mammalian MMR system is highly active in S phase [55]. Proper MMR activity during S phase is ensured by degradation of D-type cyclins [56]. Both early and late S phases show similar relative MMR protein abundance at replication forks [57]. Indeed, the MMR activity facilitates nascent DNA degradation at stalled replication forks in late S phase [58]. These studies thus indicate that MMR and cell cycle activities are coordinated to ensure that DNA is duplicated without errors before mitosis. However, if the error is on the template strand, non-productive MMR reactions are triggered. For example, O6-methylguanine (meG) lesions on the template strand preferentially pair with thymine during DNA replication and consequently generates a meG:T mismatch. Since MMR targets the daughter strand, futile repair cycles of the daughter strand cause single-stranded DNA gaps that become double-strand breaks in the second S-phase, which finally result in checkpoint activation, cell cycle arrest, and programmed cell death in humans [59]. Futile MMR cycling across meG lesions was reported to be assisted by Smarcad1 in *Xenopus* egg extracts [60]. It should be noted that repair and cell cycle roles of the MMR system require different MMR protein levels ([61] and references therein). This conclusion is based on some observations that demonstrate that partial loss of the MMR activity; whether through *MSH2* or *MSH6* missense mutations in yeast and mice or low MSH6 or MLH1/PMS2 levels in human; differentially affected each MMR function [61].

The connection between the MMR system and the cell cycle control is also conserved in plants. In the lab, we have previously demonstrated that the progression of the cell cycle is promoted in the *msh7* mutant under normal growth conditions and altered in *msh2* and *msh7* mutant plants in response to different stress conditions [2,62,63]. These findings indicate that the MMR system and the progression of the cell cycle are interconnected and regulated during growth and development.

## How is the MMR system regulated?

MMR genes have been reported to be transcriptional and post-translational regulated (Table 3). Transcription factors contributing to basal activity of *MSH* gene promoters are the specificity protein 1 (SP1) and nuclear transcription factor Y (NF-Y) in human. SP transcription factors are the major GC box-binding proteins [64]. SP1 regulates stem cell maintenance, cell proliferation, cell growth, apoptosis, and tissue differentiation [65]. An essential role of SP1 for the transcriptional regulation of human *MSH2* and *MSH6* has been reported [66]. The trimeric NF-Y transcription factor is the major CCAAT box-binding factor [64]. A polymorphism in NF-Y transcription factor binding site of human *MSH2* resulted in reduced *MSH2* expression [67]. The NF-Y is widespread in eukaryotes and is also termed heme activator protein (HAP) [68]. In *S. cerevisiae*, the Hap complex contains three essential subunits Hap2/3/5 for binding to the CCAAT box and an additional subunit Hap4 for providing an activation domain [69]. MSH2 was down-regulated in *HAP2*-deleted and *HAP4*-deleted *S. cerevisiae* strains [70,71]. Although the regulation of *A. thaliana* MMR genes has not yet been experimentally validated, predicted transcription regulation network was analyzed using https://plantpan.itps.ncku.edu.tw/plantpan4/index.html with sequences spanning from 100 bp downstream to 1000 bp upstream the transcription start site of MMR genes. Results identified NF-Y binding domains in *MSH2* (located at −147, −369, and −23 bp), *MSH3* (located at 41 and 62 bp), *MSH6* (located at 59 bp), and *MSH7* (located at −42, −248, −347, −755, −906, and −951) gene promoters. The conservation of binding motifs of these transcription factors between species highlights the important role of the MMR system in maintaining the stability of the genome.

**Table 3:** Transcriptional and post-translational regulation of the MMR system in model organisms

		MMR system						
		*S. cerevisiae*		*H. sapiens*		*A. thaliana*		References
**Regulation**	**Transcriptional**	**Hap2/Hap4**	*MSH2*	**SP1**	*MSH2* *MSH6*	**NF-Y**	? (only prediction)	[66,67, 70, 71]
		**Swi6/4-Mbp1**	*MSH2*	**E2F**	*MSH2* *MSH6*	**E2F**	*MSH3* *MSH6*	[52,54,72,73]
		**Promoter methylation**	?	**Promoter methylation**	*MSH2* *MLH1*	**Promoter methylation**	*MSH6*	[74,75]
	**Post- translational**	**Phosphorylation**	?	**Phosphorylation**	MLH1 PMS2	**Phosphorylation**	? (evidence for cotton PMS1)	[76–80]
		**Acetylation/deacteylation**	Msh2 Msh6	**Acetylation/deacteylation**	MSH2 MSH3 MLH1	**Acetylation/deacteylation**	?	[81–86,87]
		**Ubiquitination**	Msh2 Msh6	**Ubiquitination**	MSH2	**Ubiquitination**	?	[84,87]

The question mark (?) represents unknown interactions. Details are described in the text.

Transcription factors involved in the repair of DNA damage during cell cycle progression include adenovirus E2 binding factor (E2F) in human and plants. Experimental analysis validated human *MSH2* and *MSH6* and *A. thaliana MSH3* and *MSH6* as responsive to E2F transcription factors [52,54,72]. The yeast E2F family consists of SCB- (SBF, heterodimer of Swi4 and Swi6) and MCB-binding factors (heterodimer of Mbpl and Swi6) [88]. Yeast Swi6, Swi4, and Mbp1 were reported to be required for basal *MSH2* activity [73]. These data indicate that the interaction between the MMR system and the cell cycle progression is functionally conserved through evolution.

Transcription factors involved in the control of plant specific responses were demonstrated for the *MSH6* gene. In *A. thaliana, MSH6* expression was induced by light exposure, sugar or phytohormone treatments [89]. Light, sugar, or auxin responsiveness was mediated by phytochrome A, WUSCHEL, or GATA23 signalling, respectively [89].

On the other hand, transcriptional silencing of MMR gene promoters by hypermethylation is documented. Hypermethylation of human *MLH1* or *MSH2* promoter sequences caused somatic gene silencing [74], while hypermethylation of plant *MSH6* promoter sequences was observed in the progeny of salt stressed plants [75].

Post-translational modifications also regulate the MMR activity. Human MLH1 has been found to be phosphorylated at several S residues [76,77]. Phosphorylation of an S residue located in the N-terminal ATPase domain induced DNA binding loss and phosphorylation of S residues located in the linker domain induced MMR activity loss [76,77]. Human PMS2 has also been reported to be phosphorylated and phosphorylation changed protein stability [78,79]. Phosphorylated cotton PMS1 was also differentially phosphorylated in primary embryogenic calli versus non-embryogenic calli [80].

Human MMR activity is also regulated by acetylation [81]. MLH1 acetylation by the histone acetyl transferase CREB binding protein promoted MutSα–MutLα complex formation leading to an increased MMR activity [82]. On the contrary, MLH1 deacetylation by the histone deacetylase 6 (HDAC6) disrupted the MutSα–MutLα complex [83]. Interestingly, one of the K target for acetylation is conserved in *S. cerevisiae* and *A. thaliana*, among other eukaryotes [83]. MSH2 was also deacetylated by HDAC6, HDAC10, and by the sirtuin SIRT7 leading to an impaired MMR activity [84–86]. In addition, HDAC6 enables MSH2 protein degradation through the zinc-finger ubiquitin binding domain [84]. Similarly, MSH6 and MSH3 acetylation were regulated by HDAC proteins, HDAC1 and HDAC3, respectively [85,90]. In yeast, Msh2 and Msh6 levels were also modulated by acetylation and ubiquitination [87].

Overall reports indicate that several regulatory processes can influence the MMR activity.

## Why is the MMR system important?

Deficiency in the MMR system is characterized by an elevated mutation rate. In human cells, MMR deficiency predisposes to hereditary cancer syndromes such as Lynch syndrome and sporadic tumour development in human cells [91–93]. Consequently, frameshift mutations in coding regions could produce short peptides, which can function as neoantigens and generate immunogenic responses [94–96]. In eukaryotic pathogens, MMR deficiency contributes to the emergence and evolution of hypermutator phenotypes in eukaryotic pathogens [97–99], while in plants, leads to genotypic and phenotypic changes [100–102].

In addition, some MMR proteins can also act as pro-mutagenic promoting trinucleotide repeat expansions [103–105]. Abnormal lengthening of specific trinucleotide repeats can result in several neurodegenerative diseases, including Huntington’s disease, Friedreich ataxia, myotonic dystrophy type 1, and the Fragile X-related disorders [36,106].

Thus, the elevated mutation rate on one hand has been implicated in human genetic diseases but on the other hand in an enhanced development of resistance to antifungals or plant adaptation to environmental stress (Table 4 [63,102,107–110]).

**Table 4:** Effect of MMR system deficiency in different organisms

MMR system deficiency		
↓		
Mutant cell accumulation		
Fungal pathogens	Humans	Plants
Antifungal susceptibility or resistance	Disease	Harmful or advantageous traits

Details are described in the text.

## Perspectives

- The MMR system is a crucial and conserved guardian of the genome against DNA replication and recombination errors and DNA damage induced by endogenous and exogenous factors.
- Remarkable advances have been made in the mechanism, occurrence, regulation, and significance of the eukaryotic MMR system. Interesting similarities and differences between organisms have been provided. Similarities include the mechanism of action and differences comprise some aspects of regulation.
- Much has yet to be learned before our understanding is complete, especially in plants, the least studied system.

## Open Access

This article has been published open access under our Subscribe to Open programme, made possible through the support of our subscribing institutions, learn more here: https://portlandpress.com/pages/open_access_options_and_prices#conditional.

## Abbreviations

CAF-1: chromatin assembly factor-1
CHR19: CHROMATIN REMODELING19
E2F: Adenovirus E2 binding factor
EXO1: exonuclease 1
HAP: heme activator protein
HDAC6: histone deacetylase 6
HLTF: helicase-like transcription factor
IDL: insertion/deletion loop
LFR: leaf- and flower-related
meG: O6-methylguanine
MMR: mismatch repair
MLH1: MutL homolog 1
MSH: MutS homolog
NF-Y: nuclear transcription factor Y
PCNA: proliferating cell nuclear antigen
SHPRH: SNF2 histone linker PHD RING helicase
SP1: specificity protein 1
SWI/SNF: switch/sucrose-non-fermenting
